# To be or not to be an Obstetrician / Gynaecologist

**DOI:** 10.12669/pjms.36.6.3080

**Published:** 2020

**Authors:** Iffat Ahmed, Abid Ashar

**Affiliations:** 1 Iffat Ahmed, Assistant Professor, Department Obstetrics Gynaecology, The Aga Khan University, Karachi, Pakistan; 2 Prof. Abid Ashar Principal, College of Dentistry, Professor of Oral & Maxillofacial Surgery, Fatima Memorial Hospital, College of Medicine & Dentistry, Shadman, Lahore, Pakistan

**Keywords:** Career counselling, Career guidance, Motivation, Obstetrics and Gynaecology as a career, Post-graduate training

## Abstract

**Objectives:**

This qualitative study aimed to explore motivational sources of physicians, at the time of selection and while pursuing ObGyn career. Secondary aim was to explore challenges and strategies adapted by these physicians to overcome these challenges.

**Methods:**

This is qualitative study with constrictive worldview. ObGyn residents and consultants of Aga Khan University, Karachi, were interviewed from July 2017 till Jan 2019, after ethical approval, using purposeful maximum variation sampling. Analysis were conducted by identifying keywords and phrases, these unedited verbatim with no assumptions provided basis for codes, which then clustered as trends. Emerging findings were discussed among authors and themes were finalized with consensus. Conclusion was formulated by linking these themes.

**Results:**

Four themes emerged were, ‘grounds for selecting ObGyn as career’, ‘Motivational Factors’, ‘Demotivating Factors’ and ‘Strategies to Cope with Challenges’. Results showed that aptitude and passion not only have pivotal role in career selection but also helped in pursuance. Personal fulfilment and hands-on experience satisfy emotional needs, while family and friends supported participants in maintaining work-life balance and in over-coming challenges.

**Conclusions:**

Considering personal preference and aptitude at the time of career selection helps in endurance and keep motivations high, while challenges in pursuance can be overcome by strong support system.

## INTRODUCTION

Obstetrics and Gynaecology (ObGyn) is one of the highly demanding fields with obvious gender polarization.^1^ Specially in social structure like that of Pakistan, women prefer female doctor for ObGyn services. This cultural influence makes ObGyn a preferred choice of female physicians for postgraduate training.^2^ Personal aptitude and desires of family also contribute to this decision.^3^ While prolonged working hours with family commitments makes ObGyn extremely challenging, especially for young female doctors. It is often difficult for them to maintain work-life balance. Although national data is not available but dropout rate and switching to specialty, were observed among ObGyn resident. Even the residents with a diehard passion for this speciality, are sometime unable to cope with the challenges.^2^,^4^ High levels of commitment and motivation are required to deal-in with day to day challenges.

International literature has analysed various aspects of selecting ObGyn and motivational undercurrents for career pursuance, but to the best of our knowledge local studies did not focus on ObGyn physicians and most of these are descriptive studies.^5^ This qualitative study was intended to explore motivational sources at the time of selection and while pursuing ObGyn career. While, secondary aim was to explore challenges and strategies adapted by these physicians to overcome these challenges. It can serve as a career-guide for medical graduates and mentoring junior doctors. Moreover, it can be helpful in modifying post-graduate curricula in accordance with participants interests.

## METHODS

This qualitative study with constrictive worldview targeted physicians including residents and consultants of ObGyn department, Aga Khan University (AKU), Karachi Pakistan conducted from July 2017 till Jan 2019. Phenomenology Design was used for data analysis. Considering limited population and their willingness for participation, purposeful maximum variation sampling technique was used. Approval was obtained from ‘Ethical Review Committee, The Aga Khan University’ (4524-Obs-ERC-16 (Nov 21,2016). Study subjects were divided into two groups; ‘residents group’ included post-graduate trainees from year one to four and ‘consultants group’ comprised of physicians working in ObGyn. Participants were approached after seeking permission from chairperson of ObGyn department. Variation among participants was managed by including residents from each year of training, while consultants from different subspecialties, age groups and years of experience.

In-depth Interviews were conducted, with written consents of participants, until data was saturated. Semistructured predetermined interview guide was used, consisting of open-ended questions with predecided probes. Dry run of interview guide was conducted before formal interviews. Ideas were further explored depending upon responses but four basic questions were asked form each participant. Sequence of questions and alternate phrases complemented one another and maintained the dependability of responses.

The basic questions were:

- Why did you select OBGYN as a career?
- What are the factors that kept you motivated to pursue OBGYN as career?
- What were the factors that made it difficult to continue in this field?
- How did you cope with the difficulties?

Interviews were conducted in English and recorded on voice recorders. Field notes were taken by the researcher including observations of nonverbal gestures, tone and unintended responses. These field notes helped in triangulation of data and transcripts were sent back to respondent for member checking. Confidentiality was assured by maintaining participants’ anonymity and not mentioning age, year of experience subspecialty. Code ‘R’ represents residents and ‘C’ is for consultant whereas number was allotted to each participant, alphabetically. Recordings were destroyed two weeks after validation while transcript will be stored with password protection for five years as a property of AKU.

Analysis were conducted by identifying keywords and phrases, these unedited verbatim with no assumptions provided the basis for coding. Codes were clustered as trends and overarching themes were extracted from these trends. Emerging findings were discussed among authors and themes were finalized with consensus. These themes were then linked together to formulate a conclusion.

## RESULTS

All participants partake in our study were females. Post-graduation experience of consultants ranged from five to 30 years. Data got saturated after interviewing nine participants from each group. Four themes emerged after interpretation and comprehension of trends include; ‘Grounds for Selecting ObGyn as Career’, ‘Motivating factors’, (Table-I) ‘demotivating factors’ and ‘Strategies to Cope with Challenges’ (Tables-II).

**Table-I:** Selected comments and Trends of Theme I and II.

***Theme I: Grounds for selecting OBGYN as career***
***Trends:***
***1: Personal preference (04/11)***
1. ‘Actually, initially I joined dermatology …., but soon I realized that I am not a physician, I am a surgeon…. That’s why I took OBGYN …..’ (C2)
2. ‘I was the only person in top ten who applied in OBGYN willingly, ….and I enjoyed my internship’ (C4).
3. ‘I developed interest in final year then after my house job…. I want something surgical that’s why I select OBGYN’ (R2)
4. ‘Basically, reason for coming into OBGYN is just I want to go for OBGYN…. it includes medicine and surgery both’ (R7)
***2: Guidance from family/mentor (02/08)***
5. ‘….it was more acceptable to my family…. I discussed with one of my Professors….’ (C4)
6. ‘I selected OBGYN firstly because, to some extent, of family’s pressure.’ (C8)
7. ‘Actually, I didn’t select it, my family and my father wanted me to be a gynaecologist….’(R5)
***3: Personal experience (02/04)***
8. ‘…. the other reason is very personal one, my mother has had a very bad obstetric history. She had hysterectomy at the age of twenty-two….’ (C1)
9. ‘….in OBGYN department, there were very poor role models, so I thought probably I can make a difference.’(C5)
***4: Female-oriented field (02/04)***
10. ‘…. as a female I think it was a better option for me……’(C8)
11. ‘Secondly it is more female-oriented field. As compared to surgery where you have to compete with male surgeons…’ (R2)
***5: Limited syllabus (compare to other fields) (02/02)***
12. ‘…..I came into ObGyn because it has shortest syllabus’ (C1)
13.‘I have already lost six years .… I thought this is the short-term thing that I can do …’ (C2)
***6: Financial attraction (02/03)***
14. ‘Like! it was economically and financially is expected to be more productive ….’ (C9)
15. ‘….as a gynaecologist I will be able to establish early…. life style changes’ (R9)

***Theme II: Motivating factors***
***Trends***
***1: Emotional Satisfaction (04/16)***
16. ‘At the same time, it is a very fulfilling experience because I love to listen to people’s stories actually….’ (C4)
17. ‘Especially when we save a mother or see a crying cute baby, it makes us very happy’ (C8)
18. ‘It was an awesome feeling to see happiness on the mother’s face’ (C7)
19. ‘…. this was the interaction with the patients which makes me happy….’ (R3)
***2: Passion/Aptitude (04/06)***
20. ‘……once I get into ObGyn then I really started enjoying it because it has both the component of medicine as well as surgery. (C1)
21. ‘……surgery is my passion; I am performing hands-on surgery that keeps me motivated.’ (C7)
22. ‘…. that I found it a very interesting ….. I was basically made for it. ……’ (C8)
23. ‘…. it’s just the passion .…basically it gives me happiness that’s why I am interested ….’ (R1)
***3: Dealing with healthier population (02/03)***
24. ‘….. there were very less mortalities……we are giving healthy babies to healthier mothers’ (C7)
25. ‘……the good point about OBGYN is that you got healthy population …..’ (R7)
***4: Role model/mentor (01/06)***
26. ‘Maybe I am too much obsessed with the figure of my supervisor. She also had the same circumstances as mine; …. that kept me motivated’ (C2)

**Table-II:** Selected comments and Trends of Theme III & IV.

***Theme III: De-motivating factors:***
***Trends***
***1: Challenges in maintaining work-life balance (2/10)***
1. ‘…. for women who pursue professional careers, there is an element of sacrifice that they and their families have to go through …I think it is not just for OBGYN this is true for almost every professional woman …’ (C6)
2. ‘Yes! I really faced a lot of difficulties, like I started my career and married life almost simultaneously. I was working and looking after my family…. I faced a lot of difficulties…. my pregnancies went along with night on-calls. Later I had to leave my small babies at home while they were still on breast-feed and I was not able to come home to feed them. It was really the most horrible time period that I faced and that exhausted me a lot’ (C8)
***2: Long working hours (03/15)***
3. ‘….. long working hours of this field definitely sometimes make me annoyed’ (C2)
4.‘….We work day and night….people expect us to work 24/7 which is not humanly possible ….ObGyn is really tough’ (C3)
5.‘….ObGyn sometimes gets hectic, which other fields can also be.…So not specifically for the gynae….’ (R3)
***3: Negative feedback/Lack of encouragement (01/04)***
6. ‘Sometimes the feedback which we got. I think there are less people who encourage you, but more will discourage you…’ (R9)

***Theme IV: Strategies to cope with challenges***
***Trends***
***1: Support system (05/17)***
7. ‘….my daughter has helped me a lot. She has been the biggest support throughout my training.….’ (C1)
8. ‘…… obviously my husband as well but my mother was the main support …. (C2)
9. ‘…. I had a lot of family support in terms of my extended family, my husband and then later on my children…’ (C6)
10. ‘Basically, these are friends who make you relaxed….’ (R1)
11.‘…. I have made many good friends, … so like we go for shopping whenever we got time, and get together.’ (R8)
***2: Positive attitude/ healthy activities (04/08)***
12. ‘Meditation, sometimes exercising, swimming and then sometimes taking a vacations, to get some relief……’(C2)
13.‘Actually, if you truly enjoy what you do, you don’t see difficulties you see opportunities’ (C5)
14. ‘…..when everyone here, is getting through the same situation, ….so why can’t I’(R2)
15. ‘…. actually, I never blame that this is because I chose OBGYN…..I tell myself “I have to go through it even if I am not comfortable”….’ (R3)

Prevalent themes, trends within these themes, and selected comments verbatim form participants, representing different data chunks in transcripts are shown in Table-I and II. Selected comments and total responses coded together as a trend are given in parentheses (selected comments/total comments). Table-III depicts frequencies of trends among consultants and residents.

**Table-III:** Frequency of themes and trends among consultants and residents.

S. No.	Themes & Trends	Consultants’ Responses	Residents’ Responses	Total Responses
***Theme I: Grounds for selecting OBGYN as career (32 Comments)***					
1.	Personal preference	04	07	11
2.	Guidance from family/mentor	03	05	08
3.	Personal experience	04	-	04
4.	Female-oriented field	02	02	04
5.	Limited syllabus	02	--	02
6.	Financial attraction	01	02	03
***Theme II: Motivating Factors (31 Comments)***					
7.	Emotional satisfaction	08	08	16
8.	Personal aptitude	03	03	06
9.	Dealing with healthier population	02	01	03
10.	Role model/mentor	01	05	06
***Theme III: De-motivating Factors (29 Comments)***					
11.	Maintaining work-life balance	05	05	10
12.	Long working hours	06	09	15
13.	Negative feedback/Lack of encouragement	02	02	04
***Theme IV: Strategies to Cope with Challenges (25 Comments)***					
14.	Support system	05	12	17
15.	Positive attitude/ healthy activities	02	06	08

## DISCUSSION

This study focused on motivations of doctors while selecting and during pursuing career in ObGyn. It also explored demotivating factors and some strartegies adapted by our participants to overcome these challenges. Qualitative method was used, for in-depth exploration and understanding. These findings will help in career-counselling and mentoring young doctors during different phases of their career. It will be a contribution to literature in local perspective.

*Personal Preference* was the fore-most reason for career-selection. Eleven out of 18 participants either have a *passion* for ObGyn or for surgery,^6^ (Table-I, comment 1-4) while few liked the diversity of having medical and surgical component in ObGyn (Tabl-I, comment 4, 20).^7^ This attitude towards surgery is highly motivational, most of our participants enjoy hand-on surgeries and found experiential learning highly effective. (Table-I, comment 20-23).^4^,^8^-^10^ Therefore, ObGyn postgraduate curriculum should include more of psychomotor component.

Decisions of participants seemed to be influenced by families (Table-I, comment 05-07), yet, most of these respondents (14/18) appreciated the ultimate support of their kins. This may be because Pakistan has strong clanship and decisions of children specially women are mostly influenced by family, along with care and encouragement.^2^,^11^ (Table-II, comment 07-11)

Overall medical professionals are compassionate, especially female doctors are more social and connected to their patients emotionally.^9^, ^11^-^14^ Empathy and altruism was obvious among our physicians as well. Almost every participant (16/18) considered *Emotional Satisfaction* as a major motivating factor. (Table-I, comment 16-19).

A proven influence of role models and mentors was found to be a continuous source of inspiration and support during different career-stages in our study.^4^,^10^ (Table-I, comment 26) Although Higham considered ‘*lack of positive role models’* in ObGyn causing disinterest among medical students,^7^ but it compelled one of our consultants to become an ObGyn specialist. (Table-I, comment 09).

Gender polarization in ObGyn was evident as a cultural norm in this group as well. Four out of them considered it as ‘female-oriented’ field and were comfortable in dealing with female patients and co-worker.^4^,^8^,^13^,^15^-^17^ (Table-I, comment 10-11) Interestingly, in ObGyn department all but two consultants are females, while over past ten years no male doctor opted for ObGyn residency.

ObGyn, being one of the highly paid specialties, was chosen by medical students for financial benefits and paying debts.^3^,^8^,^13^,^16^,^18^ However, our results were more consistent with Scott et al., because financial aspect was not the primary motive among this group.^9^ Three out of eighteen participants have mentioned it as a secondary consideration. (Table-I comment, 14-15)

ObGyn with narrow scope of practice, may be a good choice for those who looking for a short course-line, however this doesn’t mean a less hectic job or a controllable life-style.^9^ This subliminal thought was shared by two of highly committed and busy consultants. Interestingly they both resumed their career after completing their families. (Table-I, comment 12-13)

As reported by Shirley and Hochberg, major trials for our subjects were maintaining work-life balance (Table-II, comment 1-2) and prolonged working hours (Table-II, comment 3-5).^10^,^14^,^18^ But most of our participants accepted it as a challenge and tried to manage it with positive attitude and healthy activities. (Table-II, comment 12-15) Maintaining work-life balance, according to them, would be a challenge for any working women in medical and nonmedical professions, especially if dealing-in with emergencies (Table-II, comment 01,05).

### Limitations of the study

Study focused only on ObGyn residents & consultants at a single centre where the principle investigator works. Since these are the doctors who are already committed to this profession so may not represent those who have already quit. Moreover, AKU is a private university therefore, this study may not represent the views of doctors in other hospitals of private and government sector.

## CONCLUSION

Pursuance of career needs passion, therefore, aptitude of individual should be considered during career-counselling and on induction in postgraduate programmes. Structured mentoring programmes can be introduced in ObGyn for support and guidance to overcome the potential challenges. Further research is needed, to broaden the understanding for designing ObGyn curriculum and shaping physicians’ motivations.

### Author`s Contribution:

**IA, AA** conceived, designed, analysis/interpretation of data.

**IA** did data collection/management, manuscript writing, responsible/accountable of study.

**AA** did review and final approval.
